# Segmentation for Multimodal Brain Tumor Images Using Dual-Tree Complex Wavelet Transform and Deep Reinforcement Learning

**DOI:** 10.1155/2022/5369516

**Published:** 2022-05-23

**Authors:** Gang Liu, Xiaofeng Li, Yingjie Cai

**Affiliations:** ^1^College of Computer Science and Technology, Harbin Engineering University, Harbin 150001, China; ^2^State Key Lab for Novel Software Technology, Nanjing University, Nanjing 210008, China; ^3^Department of Information Engineering, Heilongjiang International University, Harbin 150025, China; ^4^The First Psychiatric, Hospital of Harbin, Harbin 150056, China

## Abstract

Image segmentation is an effective tool for computer-aided medical treatment, to retain the detailed features and edges of the segmented image and improve the segmentation accuracy. Therefore, a segmentation algorithm using deep reinforcement learning (DRL) and dual-tree complex wavelet transform (DTCWT) for multimodal brain tumor images is proposed. First, the bivariate concept in DTCWT is used to determine whether the image noise points belong to the real or imaginary region, and the noise probability is checked after calculation; second, the wavelet coefficients corresponding to the region where the noise is located are selected to transform the noise into normal pixel points by bivariate; then, the conditional probability of occurrence of marker points in the edge and center regions of the image is calculated with the target points, and the initial segmentation of the image is achieved by the known wavelet coefficients; finally, the segmentation framework is constructed using DRL, and the network is trained by loss function to optimize the segmentation results and achieve accurate image segmentation. The experiment was evaluated on BraTS2018 dataset, CQ500 dataset, and a hospital brain tumor dataset. The results show that the algorithm in this paper can effectively remove multimodal brain tumor image noise, and the segmented image has good retention of detail features and edges, and the segmented image has high similarity with the original image. The highest information loss index of the segmentation results is only 0.18, the image boundary error is only about 0.3, and *F*-value is high, which indicates that the proposed algorithm is accurate and can operate efficiently, and has practical applicability.

## 1. Introduction

Brain tumor is a common disease that poses a serious risk to people's life and health. It is clinically proven that early and effective screening and diagnosis of the disease can improve the effectiveness of treatment. Therefore, computer image-aided diagnosis and treatment technology has been widely used in the medical field [1, 2]. Currently, various medical testing and surgical instruments, such as magnetic resonance imaging (MRI), electroencephalogram (ECG), and computed tomography imaging, are the embodiment of technological applications. In order to form medical images, these images must have high resolution and clarity to describe the pathology of various parts of a patient's body and help doctors make a diagnosis [3, 4]. The relative complexity of the brain structure makes the tumor images have more details, variable morphology, and uneven grayscale [5]. All these problems can be solved by image segmentation. A number of regions with similar properties are divided into disjoint regions to improve the uneven distribution of grayscale in the image, thus enhancing the accuracy of subsequent medical diagnosis. The study of brain tumor image segmentation technology is of great importance for diagnosis, pathway treatment, and prognosis of disease development. There are many research studies on multimodal brain tumor images at home and abroad.

Yang et al. [6] proposed a multimodal brain tumor image segmentation algorithm based on deep convolutional neural networks (DCNNs), in which the detail information in the brain tumor image was first collected, then divided into different datasets according to the feature attributes, and DCNN was used to realize the feature distribution for different datasets, respectively, and the segmentation was completed by combining the distribution characteristics. However, this algorithm does not consider noise points in the original image, which leads to low accuracy of subsequent segmentation. Dhar and Kundu [7] used a segmentation algorithm based on fuzzy set with weak continuous constraint theory; the fuzzy feature set was initially established based on the features of pixel points in the image, and the segmentation was performed based on the pixels with consistent correlation by combining the theory that the pixels with consistent feature expression in the image generally have weak correlation phenomenon. However, this algorithm ignores the diversity of pixel points in the initial feature determination of the algorithm, which leads to low accuracy and affects the subsequent segmentation accuracy. Dissanayake et al. [8] proposed a triple deep learning architecture; first, a classifier was constructed with DCNN in the study; then the classified images were localized to obtain the tumor regions of interest, and finally, the contours of the tumor boundaries were segmented centrally; however, this method leads to more information loss. Zhou et al. [9] proposed a new brain tumor segmentation algorithm, in which the individual representations generated by each encoder were used to estimate the independent parameters of the modality; then, the correlation model converted all the individual representations into potential multisource correlated representations; finally, the correlated representations across modalities were fused into shared representations by an attention mechanism; however, this method has a relatively long meteorite time. Sun et al. [10] used a multipath architecture for feature extraction, where a 3D dilated convolution is used in each path to extract different feature perceptual domains. The method evaluates the one-way model and the key components of the model through an effective set of training schemes, analyzes how these alternatives affect the performance of the experiment, and effectively accomplishes image segmentation. However, there is still a high level of error. Dutta et al. [11] used deep learning algorithms to accurately localize and characterize tumors from clinical MRI images to evaluate the sensitivity of radiomic features to tumor boundaries; the method tests five network architectures and shows good performance. However, time consuming is long. Roy et al. [12] included triple-negative breast cancer patients in an ongoing co-clinical imaging trial and generated tumor xenografts from triple-negative breast cancer patients with subtype matched to investigate the best co-clinical MRI radiomic features. The method generated multiple sets of images with different signal-to-noise ratios and used an image-independent patch-based method to measure the noise level, extracting more accurate image radiological features. However, the accuracy of the analysis of image boundaries was not high and had some errors.

Wavelet transform is an ideal tool for image quality enhancement and a conventional image processing method [13]. Deep reinforcement learning (DRL) combines the advantages of deep learning feature extraction and reinforcement learning strategy learning. It has significant advantages in image processing and other fields. It is a very popular learning technology at present [14]. However, existing DRL methods often use deeper and wider architectures for image processing to improve image quality and extract image features, resulting in high computational effort and large sample data requirements. To address the shortcomings of existing studies, since multimodal brain tumor images are easily affected by various interference factors, it is difficult for the traditional algorithm to obtain better segmentation results. In this paper, we combine wavelet transform and DRL to propose a multimodal brain tumor image segmentation algorithm-based dual-tree complex wavelet transform **(**DTCWT) and DRL; the image is first preprocessed, then a sequence of feature transformations is established, and the scale value of each pixel point in the original image is calculated according to the Gaussian model, using the feature energy as the initial reference value of the segmentation function. Initial segmentation is achieved according to the scale criteria. Then, a DRL segmentation framework is constructed and a loss function training network is built to complete segmentation for multimodal brain tumor images. The results show that the proposed algorithm has better segmentation effect and outperforms the traditional algorithm in terms of similarity, information loss, boundary error, and running time. The main contributions of this paper are as follows: (1) The concept of wavelet transform was used to precapture, analyze the noise points in the images, calculate the posterior probability, and remove the image noise by transformation. (2) Image features were utilized and the features were used as a basis for segmentation to improve the accuracy of segmentation. (3) The noise challenge was solved by the preprocessing step, making the segmentation process simple to implement and effective. (4) Based on the initial segmentation results of wavelet transform, further segmentation is performed by DRL, which reduces the amount of sample data and computation, and optimizes the segmentation results to improve the segmentation accuracy.

## 2. Methodology

### 2.1. Dataset

The data used for the experiments were obtained from the BraTS2018 dataset, the CQ500 dataset, and A hospital brain tumor dataset. BraTS2018 dataset is a MICCAI competition dataset with 285 cases and 5 categories of labels: healthy brain tissue, necrotic areas, edematous areas, enhanced and nonenhanced areas of the tumor. The dataset contains MRI images before and after enhancement. CQ500 dataset: it contains head CT (electron computed tomography) images containing mass effect, hemorrhage. It contains 491 scanned images and nearly 200,000 slices, which is suitable for brain tumor segmentation studies. A hospital brain tumor dataset: it contains CT images of 100 patients with brain diseases, including brain tumor and brain hemorrhage, and also contains a large number of related clinical parameters. CT images of brain tumors from 50 cases were selected in BraTS2018 dataset, CQ500 dataset, and a hospital brain tumor dataset, respectively, to extract image information; 70% of the data are used for algorithm training and 30% of the data are used for algorithm testing. CT images of brain tumors in three datasets were used as experimental objects for detailed segmentation of multimodal tumor images, and were compared and analyzed with Yang et al. [6], Dhar and Kundu [7], Dissanayake et al. [8], Zhou et al. [9], and Sun et al. [10] to determine the effectiveness of the proposed algorithm.

### 2.2. Evaluation Criteria

Since tumor images selected from different datasets show different detailed features, there is a limitation to detect only segmentation accuracy. Therefore, the experiment compares five evaluation criteria by segmenting image effect, similarity index, information loss index, boundary error, and *F*-value in order to accurately verify the necessity of the proposed algorithm.

(1) Comparison of segmented images: select sample images to be segmented by different algorithms and compare the sharpness and noise content of the segmented images.
(2) Similarity Index: it is used to determine whether specific attributes in the segmented image are consistent with the original image [15]. The detection region is denoted as *F*. For a pixel *γ*_*i*_ and *γ*_*j*_, the similarity index determines whether the two belong to the same feature class; the higher the index value, the stronger the consistency and the better the segmentation effect. The calculation equation is
  (1) $$\begin{array}{c} \chi \left(\gamma_{i},\gamma_{j}\right)=\left\{\begin{array}{cc} 1, & \text{if }\gamma_{ij}=\gamma_{i^{\prime }j^{\prime }},\\ 0, & \text{if }\gamma_{ij}\neq \gamma_{i^{\prime }j^{\prime }}, \end{array}\right. \end{array}$$
where, *γ*_*i*′*j*′_ denotes the segmentation node corresponding to the original node; *χ*(*γ*_*i*_, *γ*_*j*_) denotes the sampling function. The segmentation result is denoted as *ℜ*^2^ and described by the set *ℜ*^2^={*r*_11_, *r*_12_,…, *r*_*mn*_}. After comparative analysis of the results to be measured and the reference results, the following equation can be obtained:
  (2) $$\begin{array}{c} \xi \left(F,{ℜ}^{2}\right)=1-\frac{1}{\left[n/2\right]}\sum_{i,j}\chi \left(\gamma_{ij},\gamma_{i^{\prime }j^{\prime }}\right)-\chi \left(\gamma_{i^{\prime }j^{\prime }},\gamma_{i^{\prime }j^{\prime }}\right), \end{array}$$
where *ξ* represents the similarity value; the larger the value, the higher the consistency between two nodes and the better the segmentation effect.
(3) Information loss index [16]: from a measurement perspective, to reflect the amount of information loss after segmentation by different algorithms,
  (3) $$\begin{array}{c} \text{PRI}\left(F,{ℜ}^{2}\right)=D^{2}\left(F\right)+L^{2}\left({ℜ}^{2}\right)-2I\left(F,{ℜ}^{2}\right), \end{array}$$
where *D*^2^ denotes the segmentation metric. *I*(*F*, *ℜ*^2^) is the common information contained between *F* and *ℜ*^2^. The smaller the value of PRI, the less information is lost in the segmented image and the better the segmentation effect.
(4) Boundary Error: it is used to detect the edge pixel distance between the segmented image and the original image. The smaller the distance value, the smaller the boundary difference between the two, and the better the segmentation effect.
(5) The *F*-value: the *F*-value is the weighted summed average of recall and accuracy, and a higher *F*-value indicates a more effective experimental method. The calculation equation is
  (4) $$\begin{array}{c} F-\text{Measure}=\frac{2\cdot \text{Pre}\cdot \text{Rec}}{\text{Pre}+\text{Rec}}, \end{array}$$

where Pre is the accuracy and Rec is the recall.

### 2.3. The Proposed Algorithm

#### 2.3.1. DTCWT-Based Image Denoising

Before segmentation of specific multimodal brain tumor images, the noise problem existing in the original images needs to be solved using the DTCWT algorithm [17] so that the conversion of noisy and non-noisy pixel points can be achieved based on the bivariate concept while retaining the original features to the maximum extent. The bivariate model function [18] is

(5) $$\begin{array}{c} \left\{\begin{array}{c} \beta_{1}=\alpha_{1}+\delta_{1},\\ \beta_{2}=\alpha_{2}+\delta_{2}, \end{array}\right. \end{array}$$

where *β*_1_ and *β*_2_ denote wavelet transform coefficients in the same direction. *α*_1_ and *α*_2_ are the wavelet transform coefficients in different directions. *δ*_1_ and *δ*_2_ represent the real and void area in the image, respectively.

We suppose that the observation vector in an image with Gaussian white noise is calculated as

(6) $$\begin{array}{c} \beta =\alpha +\delta , \end{array}$$

where *β* denotes the complex wavelet coefficients of the observed image. *α*is the complex wavelet coefficients of the original image. *α*=(*α*_1_, *α*_2_), *β*=(*β*_1_, *β*_2_), *δ*=(*δ*_1_, *δ*_2_) is the noise vector coefficient.

(7) $$\begin{array}{c} \alpha =\left(\alpha_{1},\alpha_{2}\right),\\ \beta =\left(\beta_{1},\beta_{2}\right),\\ \delta =\left(\delta_{1},\delta_{2}\right). \end{array}$$

According to the value of *β* obtained above, the value of noise posterior probability [19,20] in *α* is deduced:

(8) $$\begin{array}{c} \alpha^{\prime \prime }\left(\beta \right)=\underset{\alpha }{\arg\max}\left[P_{\delta }\left(\beta |\alpha \right)P_{\alpha }\left(\alpha \right)\right],\\ \beta^{\prime \prime }\left(\alpha \right)=\underset{\alpha }{\arg\max}\left[P_{\delta }\left(\beta -\alpha \right)P_{\beta }\left(\beta \right)\right], \end{array}$$

where *P*_*α*_(*α*) denotes the noise probability of the original image, *P*_*β*_(*β*) denotes the noise probability of the observed image. $P_{\delta }\left(\alpha^{\prime \prime }\right)=\left(3/2\pi \varepsilon^{2}\right)\exp\left(\sqrt{3}/\varepsilon^{2}\sqrt{\alpha_{1}^{2}+\alpha_{2}^{2}}\right)$ is the priori probability density of the original image. *P*_*δ*_(*β|α*) is the noise probability density difference.

The prerequisite for finding the posterior probability using this algorithm is that the noise probability density difference *P*_*δ*_(*β* − *α*) should be known and there is a fitting relationship between it and the priori probability density *P*_*δ*_(*β|α*) of the original image. In this way, the distribution of the noise vector values can be described by the joint function [21].

(9) $$\begin{array}{c} P_{\delta }\left(\alpha^{\prime \prime }\right)=\frac{3}{2\pi \varepsilon^{2}}\exp\left(\frac{\sqrt{3}}{\varepsilon^{2}}\sqrt{\alpha_{1}^{2}+\alpha_{2}^{2}}\right), \end{array}$$

where *P*_*δ*_(*α*^″^) denotes the joint probability density function and *ε*^2^ refers to the noise variance value of the original image to be calculated.

It is assumed that the initial states of the noise in the image are independently distributed. However, they obey Gaussian distribution after wavelet transform, and the probability density *P*_*N*_(*β* − *α*) is

(10) $$\begin{array}{c} P_{N}\left(\beta -\alpha \right)=\frac{3}{2\pi \varepsilon_{N}^{2}}\exp\left(-\frac{\left(\beta_{1}-\beta_{1}{}^{2}\right)+\left(\beta_{2}-\beta_{2}{}^{2}\right)}{2\varepsilon_{N}^{2}}\right)P_{\delta }\left(\alpha^{\prime \prime }\right), \end{array}$$

where *ε*_*N*_^2^ denotes the wavelet variance value of the transformed noise. Based on this, the state value of the noise coefficient in the solid part of the region can be deduced as

(11) $$\begin{array}{c} {\hat{\delta }}_{1}=\frac{\left|\sqrt{\beta_{1}{}^{2}+\beta_{2}{}^{2}}-\sqrt{3\varepsilon_{\delta }^{2}}/\delta \right|}{\sqrt{\beta_{1}{}^{2}+\beta_{2}{}^{2}}}\beta_{1}P_{N}\left(\beta -\alpha \right), \end{array}$$

where the state values of the noise coefficients in the imaginary part of the region are

(12) $$\begin{array}{c} {\hat{\delta }}_{2}=\frac{\left|\sqrt{\beta_{1}{}^{2}+\beta_{2}{}^{2}}-\sqrt{3\varepsilon_{n}^{2}}/\delta \right|}{\sqrt{\beta_{1}{}^{2}+\beta_{2}{}^{2}}}\beta_{2}P_{N}\left(\beta -\alpha \right). \end{array}$$

According to (11), an effective conversion can be performed based on the state value of the noise, converting noise-valued pixels to normal pixels and reducing the error of subsequent segmentation. A DTCWT denoising model [22] is developed as

(13) $$\begin{array}{c} \left\{\begin{array}{c} \frac{\partial I}{\partial t}=\text{div}\left[c\left(\left\|\nabla I\right\|\right)\cdot \nabla I\right]\left({\hat{\delta }}_{1}+{\hat{\delta }}_{2}\right),\\ I\left(t=0\right)=I_{0}, \end{array}\right. \end{array}$$

where *di*  *v* denotes the noise dispersion factor. ∇ stands for the gradient factor. ‖∇*I*‖ is the noise diffusion amplitude. *c*(‖∇*I*‖) denotes the noise reduction factor. *I* is the denoising function. *I*_0_ denotes the threshold value. When the detected noise value exceeds this value, it is necessary to adjust the noise reduction factor to transform the noise reduction process, and the noise can be removed while preserving the edge features.

#### 2.3.2. Extraction of Brain Tumor Features

As an integral part of the segmentation process, feature extraction helps find the key segmentation targets in the original image [23]. After the above DTCWT denoising, the distribution of feature points in the image is irregular and large in scale due to the effect of transformation, which makes it more difficult to perform feature extraction. Based on the wavelet transform matrix, the eigenvalue changes of each part of the image at different scales were identified by gradually decreasing the matrix dimension so as to cluster the pixel points with equal features for convenient extraction and management, to improve efficiency appropriately when performing the next segmentation.

The number of clusters is determined by the kind of features contained in the image, in which *u*_1_, *u*_2_,…, *u*_*n*_ denote the sampling points. In this study, all the feature points in the image were defined as the square root of the wavelet coefficient, where the number of features of all pixel points was equal to the number of clusters; each cluster was considered as a separate eigenvalue. The extraction steps are as follows:

(1) Sample to obtain a discrete signal as *U*.
(2) Obtain the coefficient matrix *B* after performing DTCWT on the *U* value.
(3) Cluster the coefficient matrix by performing feature clustering, using *v*_1_, *v*_2_,…, *v*_*n*_ to represent the feature classes; the energy value of each class feature is derived as
  (14) $$\begin{array}{c} V\left(u_{i}\right)=\sum_{i}u_{i}v_{i}^{2}, \end{array}$$

where *v*_*i*_^2^ is the feature element in the wavelet coefficient matrix corresponding to the *i* class. From the above process, it can be seen that each feature represents a set of wavelet coefficients after clustering each feature, which means that it expresses the time domain and frequency domain information of the discrete signal in the image, and describes the image features at different scales.

#### 2.3.3. Preliminary Segmentation for Multimodal Brain Tumor Images Using DTCWT

Segmentation target is an important concept in segmentation algorithm; the segmentation target is the one-to-one correspondence between the observed value of each wavelet coefficient and the actual segmentation value obtained in the DTCWT. The segmentation target is denoted as (*B*_*s*_, *E*_*s*_). The conditional probability is *P*(*B*_*s*_, *E*_*s*_), which represents the dependency between wavelet coefficient and segmentation value. The target point of the edge position in the image is labeled by *P*(*E*_*s*_), and *P*(*B*_*s*_) represents the priori probability of the edge target point. Under the condition that the wavelet coefficient *B*_*s*_ has been obtained, the probability of a target point occurring in the region to be segmented is

(15) $$\begin{array}{c} P\left(E_{s}|B_{s}\right)=\frac{P\left(B_{s}|E_{s}\right)\cdot P\left(E_{s}\right)}{P\left(B_{s}\right)}, \end{array}$$

There is a mutual constraint between the observed eigenvalue and the actual value of the pixel points that have been labeled during the segmentation process; based on this, its mis-segmentation rate *P*_*e*_ is obtained as

(16) $$\begin{array}{c} P_{e}=\prod_{i=0}^{J-1}P\left({\hat{E}}_{i}\neq E_{i}|B_{i}\right)\\ =1-\prod_{i=0}^{J-1}P\left(E_{i}\right)\cdot P\left(B_{i}|E_{i}\right), \end{array}$$

where ${\hat{E}}_{i}$ denotes the actual segmentation value of the edge target point *i*. *J* − 1 is the number of segmentation blocks. In a set of regions to be segmented, the mis-segmentation rate of the image needs to be minimized.

Based on the known observation complex wavelet coefficient *B*_*s*_, the maximum segmentation result can be obtained as follows:

(17) $$\begin{array}{c} \hat{f}=\arg\underset{f}{\max}\left\{P\left(c|f\right)P\left(f\right)\right\}\\ =\arg\underset{f}{\max}\left\{\prod_{n=0}^{J-1}\underset{\left(i,j\right)\in S^{\left(n\right)}}{∐}P\left(c_{ij}^{\left(n\right)}|f_{ij}^{\left(n\right)}\right)\right\}\times P\left(c_{ij}^{\left(n\right)}|f_{ij}^{\left(n\right)}\right), \end{array}$$

where *f* is the image block segmentation value, *c* is the image eigenvalue, *S*^(*n*)^ is the image block marker region, and (*i*, *j*) is the image pixel coordinate.

Equation (16) can be equated to the minimization energy problem by considering the full energy of the segmented image as the sum of the marker energy and the characteristic energy.

(18) $$\begin{array}{c} {\hat{f}}_{ij}^{\left(n\right)}=\arg\underset{f}{\max}\left\{\prod_{n=0}^{J-1}\underset{\left(i,j\right)\in S^{\left(n\right)}}{∐}\left[K\left(c_{ij}^{\left(n\right)}|f_{ij}^{\left(n\right)}\right)+K\left(c_{ij}^{\left(n\right)}|f_{ij}^{\left(n\right)}\right)\right]\right\}, \end{array}$$

where *K*(*f*_*ij*_^(*n*)^) is the marker energy. *K*(*c*_*ij*_^(*n*)^*|f*_*ij*_^(*n*)^) is the characteristic energy.

#### 2.3.4. DRL-Based Segmentation Optimization

Input: sample image and test image of multimodal brain tumor, preprocessing of the image to obtain the function values *I* of the DTCWT denoising model, and preliminary segmentation of multimodal brain tumor images using DTCWT.

Output: segmentation results of multimodal brain tumor images.

The parameters involved in the brain tumor image segmentation process are initialized, a segmentation framework is constructed based on DRL, the brain tumor image segmentation results are optimized, and the detailed process is.

**Figure 1:**
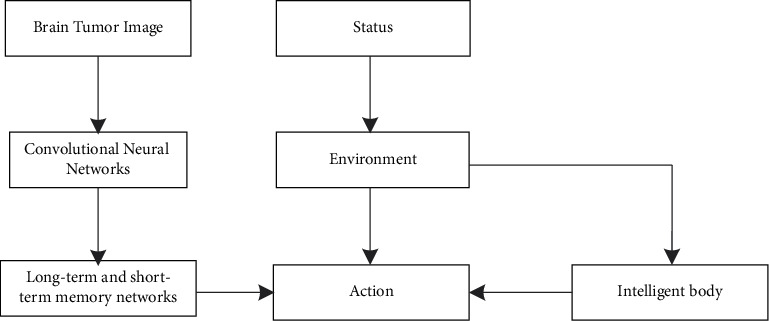
DRL-based segmentation framework.

(1) Build a multimodal brain tumor image segmentation framework using DRL, as shown in Figure 1.
According to Figure 1, during multimodal brain tumor image segmentation based on DRL, the intelligent body continuously searches for actions and related parameters based on the state of the environment; the environment gets the reward value based on the selected actions and updates the state parameters; thus, multimodal brain tumor image segmentation relies on the interaction between the intelligent body and the environment to form a decision problem.
(2) The energy values obtained from the initial segmentation of the image are used as the input vectors for the DRL model.
(3) The neural network is updated iteratively to obtain the optimal hidden layers and number of units. The DRL network is trained using the input sample vector; the training is completed based on the policy optimization algorithm. The training losses include valuation losses, action policy losses, and canonical terms. The valuation loss calculation equation is
  (19) $$\begin{array}{c} L^{V}\left(\lambda \right)=\frac{1}{2}\left(V\left(\lambda ,p\right)-R\right), \end{array}$$
where *λ* is the strategy parameter, *V*(*λ*, *p*) denotes the valuation network value function, and *R* denotes the cumulative return.
The action strategy loss is calculated as
  (20) $$\begin{array}{c} L^{\text{Action}}\left(\lambda \right)=\min\left(r_{t},k_{t}\right)r_{t}\in \left(1-\sigma \Delta 1+\sigma \right), \end{array}$$
where *r*_*t*_ denotes the probability ratio of the strategy at the time *t* to the strategy at the previous time, *k*_*t*_ is the coincidence between the values of the value function and the expected return, and *σ* is the range of values of *r*_*t*_.
The canonical term is a balance between the decision making ability and the search process. It is calculated as
  (21) $$\begin{array}{c} L^{\prime }\left(\lambda \right)=\underset{a}{\sum }O\left(a,\lambda \right)\log\;P\left(a,\lambda \right), \end{array}$$
where *O*(*a*, *λ*) indicates the output value of the action network.
The above equations are combined to complete the DRL network training and update the policy parameters and output.
(4) The data samples to be tested are input into a DRL segmentation model to complete optimal segmentation of multimodal brain tumor images and achieve segmentation of images.

**Figure 2:**
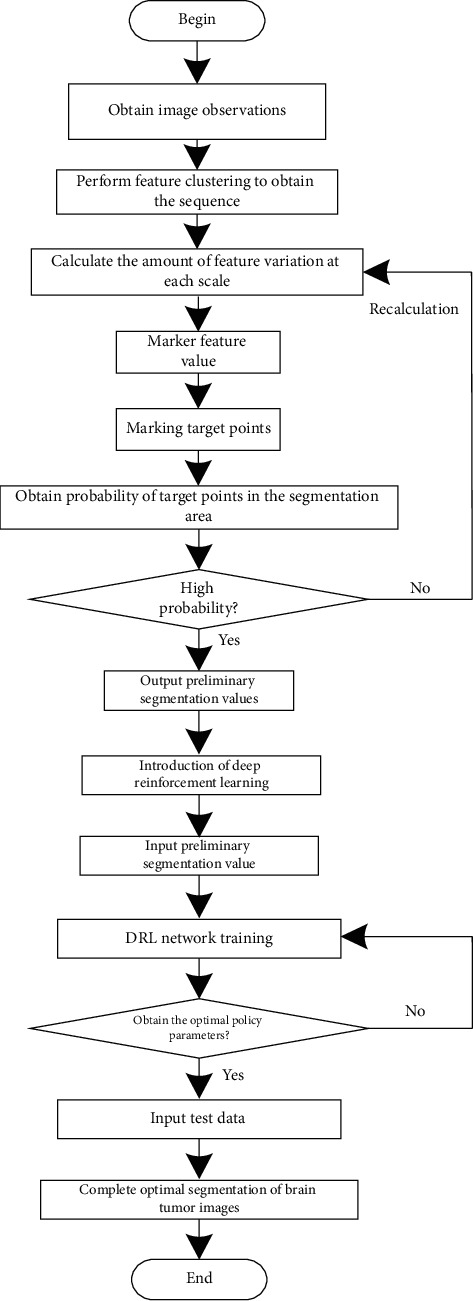
Segmentation algorithm process of multimodal brain tumor images.

The process of multimodal brain tumor image segmentation algorithm based on DTCWT and DRL is shown in Figure 2.

## 3. Results and Discussion

**Figure 3:**
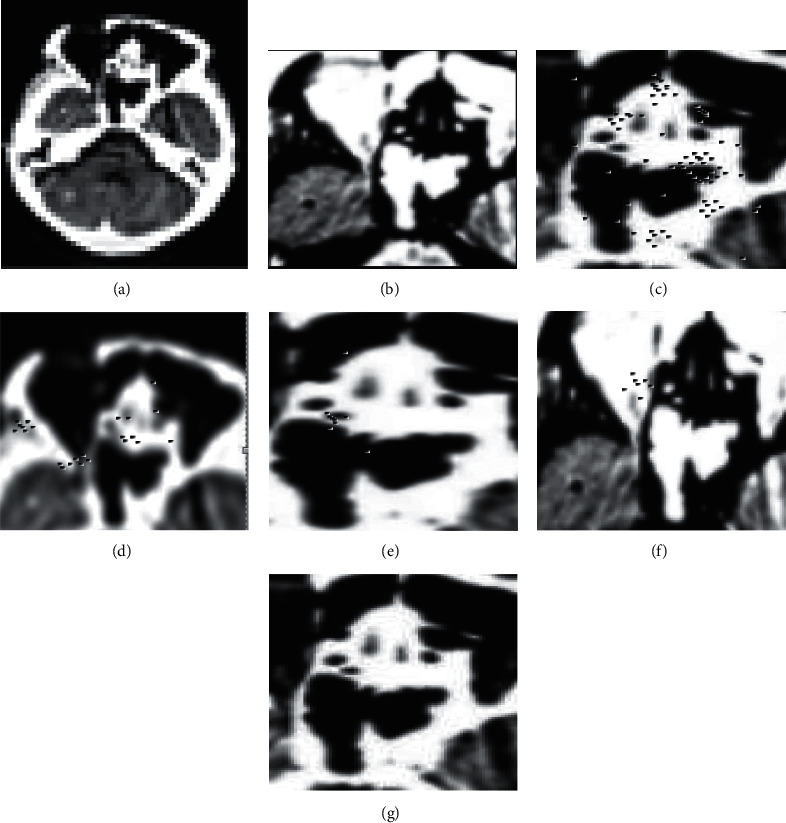
Sample image of brain tumor1. (a)Original image. (b) Yang et al. [6]. (c) Dhar and Kundu M. K [7]. (d) Dissanayake et al. [8]. (e) Zhou et al. [9]. (f) Sun et al. [10]. (g) The proposed.

**Figure 4:**
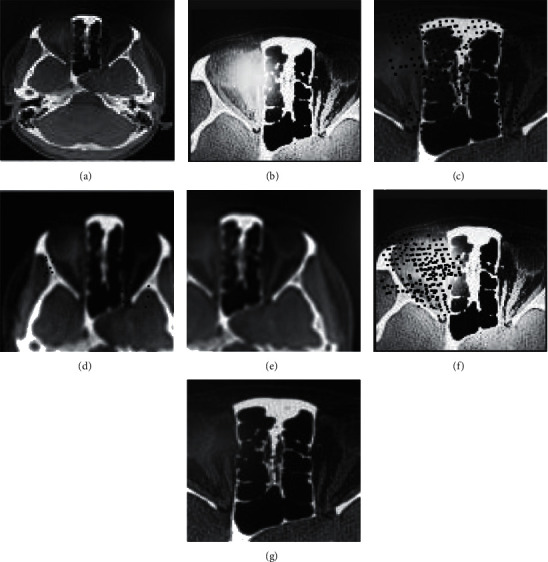
Sample image of brain tumor2. (a) Original image. (b) Yang et al. [6] (c) Dhar and Kundu [7] (d)Dissanayake et al. [8]. (e) Zhou et al. [9] (f) Sun et al. [10] (g) The proposed.

One brain tumor image was selected in each of the BraTS2018 dataset and CQ500 dataset. To improve the reference value of the segmentation results, two types of brain tumors, glioma cell and ependymoma, were used in this study because the cystic changes in both are very obvious and easy to analyze and observe; the results are shown in Figures 3 and 4. (a) is the original image, (b), (c), (d), (e), (f), and (g) are the segmented detailed images in Figures 3 and 4.

As can be seen from the example Figures 3 and 4, the brain tumor shapes in both original images are regular with clear boundary expression. After observing the segmentation results of the six algorithms, we found that the tumor detail images segmented by the proposed algorithm has the highest definition, the highest resolution, and clear edge contours, without losing the original detail features. Therefore, its segmentation effect is good. In contrast, the images segmented by the other five algorithms are blurred with low resolution and a large number of noise points; the tumor features are not clearly expressed; the details are seriously lost and distorted. This is because the proposed algorithm achieves resegmentation of the image by DRL based on the completion of the initial segmentation of the image. Therefore, the proposed algorithm obtains better segmentation results.

**Figure 5:**
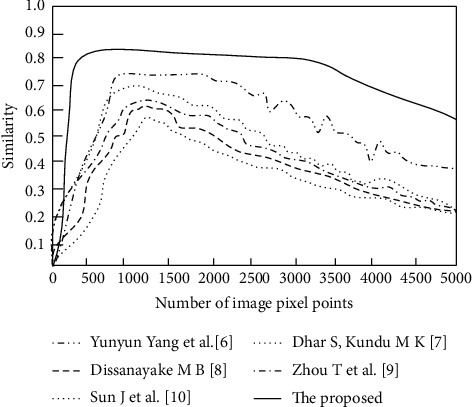
Comparison results of similarity indexes.

The image similarity comparison results of the six algorithms are shown in Figure 5.

According to comparison results of similarity indexes in Figure 5, the similarity value obtained with the proposed algorithm is the highest among all the algorithms, which is above 0.8, indicating that the segmented image with the proposed algorithm has the strongest agreement with the original image and the closest eigenvalue. As can be seen from Figure 5, after the number of pixel points is increased to 200, the image similarity value of the proposed algorithm is always higher than that of other literature algorithms, and it shows significantly higher than that of other literature algorithms at each pixel point; especially, when the number of pixel points is 500 and 3000, the similarity curve of the proposed algorithm shows two small peaks with significant advantages. In contrast, the similarity values obtained with the other algorithms are lower than those of the original image, in which, the similarity of Yang et al. [6] is relatively high above 0.7, the similarity of Dhar and Kundu [7] is also close to 0.7, the highest similarity of the algorithms of the Dissanayake et al. [8] and Zhou et al. [9] is above 0.6, and Sun et al. [10] has the lowest and does not exceed 0.6. The feature quantity of these algorithms differs greatly from the original image, indicating that the images segmented by the algorithms do not retain the original information well and are less effective. The proposed algorithm performs a detailed eigenvalue calculation and analysis of the brain tumor image before segmentation, which improves the image similarity.

**Figure 6:**
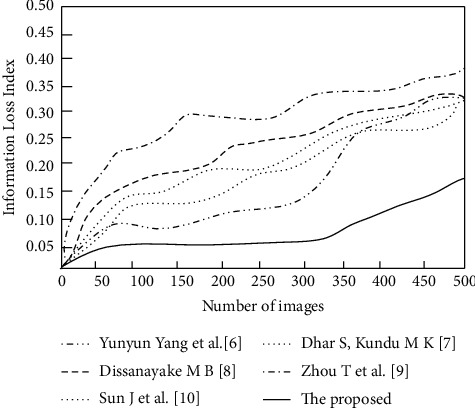
Comparison results of information loss index.

The results of comparing the information loss index of the six algorithms are shown in Figure 6.

According to the data in Figure 6, all six algorithms have more or less information loss, in which the proposed algorithm has a relatively low degree of information loss. With the increase of image information, the highest information loss index of the proposed algorithm is only 0.18. However, the information loss index of the algorithms of Yang et al. [6], Dhar and Kundu [7], Dissanayake et al. [8], and Zhou et al. [9] reaches 0.30 when the amount of image information is 500; the information loss of the algorithm of Sun et al. [10] is severer, and the information loss index is 0.37 at an image information level of 500. As shown in Figure 6, the information loss index curves of the proposed algorithm are lower than other literature algorithms at each image information amount, which has a significant advantage. Compared to the original image, this is the level that does not affect the perception and practical application detection. The reason why the proposed algorithm can well ensure the information integrity is that the noise of the image is analyzed and processed in advance during the specific segmentation so as to ensure that the key features can be extracted in the follow-up, reduce the impact of noise, and ensure the integrity of details.

**Figure 7:**
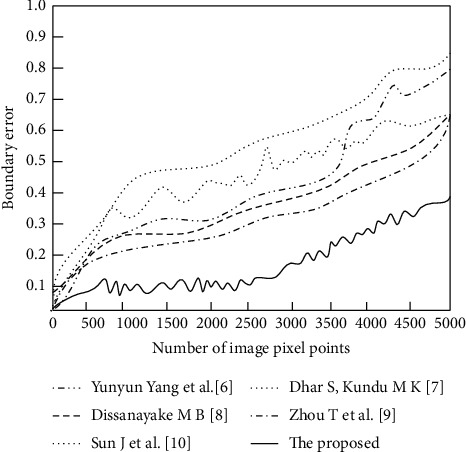
Comparison results of boundary error index.

The comparison results of the boundary errors of the six algorithms are shown in Figure 7.

According to the data in Figure 7, the boundary error indexes of the six algorithms vary widely, in which the boundary error of the algorithms in Yang et al. [6] and Sun et al. [10] is as high as about 0.8, and the highest boundary error of the algorithms in Kundu [7], Dissanayake et al. [8], and Zhou et al. [9] is 0.6, while the proposed algorithm is lower, with a boundary error of only about 0.3. Obviously, the proposed algorithm presents better experimental results for multimodal brain tumor image segmentation based on DTCWT, which has certain accuracy and practicality. Therefore, in this paper, the initial segmentation of multimodal brain tumor images is firstly performed by using DTCWT; then, the segmentation results are optimized based on DRL, and good experimental results are presented, which has certain accuracy and practicability.

The results of the *F*-value comparison of the six algorithms are shown in Table 1.

According to the data in Table 1, the *F*-values of the six algorithms for multimodal brain tumor image segmentation all keep changing with the number of image pixel points. However, the *F*-values of the algorithms in literature [6], literature [7], literature [9], and literature [10] are always below 0.80, and the *F*-values of the algorithm in literature [8] reach up to 0.86. However, it is much lower than the proposed algorithm, and the F-values of the proposed algorithm are always above 0.90 and even up to 0.96 between 1000 and 5000 pixel points. The F-value of the proposed algorithm is always above 0.90 and even up to 0.96 between 1000 and 5000 pixel points, which shows that the proposed algorithm has significant advantages and fully verifies the advantages of combining DTCWT and DRL.

## 4. Conclusions

In this paper, a segmentation algorithm using DTCWT and DRL for multimodal brain tumor images was proposed, and the wavelet transform method was used to address the large amount of noise interference in the original image. First, the region where the noise exists was acquired and analyzed to determine whether the noise points of an image belong to solid area or void area. Second, the pixel points with the same noise posterior probability were calculated. The feature extraction is carried out by sequence tagging. Then, the clustering of features at different scales was calculated, the conditional probability of segmentation of preset points in the image at edge position and center position was calculated, and the maximum segmentation value was found. The best segmentation degree was found by the dependency between wavelet coefficient and segmentation value. Based on this, the image segmentation was further performed using a DRL model to further improve the accuracy of the segmentation results. The image obtained by the proposed algorithm has a certain degree of robustness, and the acquired image has a high similarity with the original image. Therefore, the proposed algorithm can provide important contribution for medical diagnosis when applied to actual tumor image segmentation. Although certain results have been achieved in this study, there are still some technical blind spots. Further work is needed for segmentation in complex cases, especially for the segmentation of image rough edges. In future study, more relevant detailed data and parameters will be analyzed in depth to enhance the analysis of image rough edges to improve in more segmentation details.

## Figures and Tables

**Table 1:** Comparison of *F*-value results.

Image pixel point number	Yang et al. [6]	Dhar and Kundu [7]	Dissanayake et al. [8]	Zhou et al. [9]	Sun et al. [10]	The proposed
1000	0.63	0.65	0.86	0.62	0.63	0.94
2000	0.63	0.75	0.82	0.63	0.69	0.95
3000	0.65	0.69	0.85	0.69	0.65	0.96
4000	0.72	0.70	0.79	0.70	0.72	0.93
5000	0.59	0.71	0.76	0.75	0.73	0.91

## Data Availability

The data used to support the findings of this study are available from the corresponding author upon request.
